# Communication about genetic testing with breast and ovarian cancer patients: a scoping review

**DOI:** 10.1038/s41431-018-0310-4

**Published:** 2018-12-20

**Authors:** Chris Jacobs, Christine Patch, Susan Michie

**Affiliations:** 1 Graduate School of Health, University of Technology Sydney, PO Box 123, Broadway, NSW 2007 Australia; 2 Department of Clinical, Education and Health Psychology, University College London, 1-19 Torrington Place, London, WC1E 7HB UK; 3 Florence Nightingale Faculty, Nursing, Midwifery and Palliative Care, King’s College London, 57 Waterloo Road, London, SE1 8WA UK; 4 Genomics England, Queen Mary University of London, Dawson Hall, London, EC1M 6BQ UK

**Keywords:** Genetic testing, Genetic counselling

## Abstract

Genetic testing of patients with cancer is increasingly offered to guide management, resulting in a growing need for oncology health professionals to communicate genetics information and facilitate informed decision-making in a short time frame. This scoping review aimed to map and synthesise what is known about health professionals’ communication about genetic testing for hereditary breast and ovarian cancer with cancer patients. Four databases were systematically searched using a recognised scoping review method. Areas and types of research were mapped and a narrative synthesis of the findings was undertaken. Twenty-nine papers from 25 studies were included. Studies were identified about (i) information needs, (ii) process and content of genetic counselling, (iii) cognitive and emotional impact, including risk perception and recall, understanding and interpretation of genetic test results, and anxiety and distress, (iv) patients’ experiences, (v) communication shortly after diagnosis and (vi) alternatives to face-to-face genetic counselling. Patients’ need for cancer-focused, personalised information is not always met by genetic counselling. Genetic counselling tends to focus on biomedical information at the expense of psychological support. For most patients, knowledge is increased and anxiety is not raised by pre-test communication. However, some patients experience anxiety and distress when results are disclosed, particularly those tested shortly after diagnosis who are unprepared or unsupported. For many patients, pre-test communication by methods other than face-to-face genetic counselling is acceptable. Research is needed to identify patients who may benefit from genetic counselling and support and to investigate communication about hereditary breast and ovarian cancer by oncology health professionals.

## Introducton

The increase in genetic testing shortly after diagnosis to guide breast and ovarian cancer management requires a shift in pre-test genetic communication, from specialist genetics to mainstream oncology services. Oncology health professionals are increasingly required to discuss the options and possible outcomes of genetic testing with patients. Alongside this change, genetics health professionals are required to counsel cancer patients who are newly diagnosed or receiving palliative care about the implications of a genetic test result for themselves and their families. Learning from previous practice and research can help to inform the development of new approaches to communicating about genetic testing with cancer patients.

This scoping review aimed to summarise and map the range, extent and nature of the published research into communication about hereditary breast and ovarian cancer between genetics and oncology health professionals and patients. The purpose of the review was to summarise and disseminate the research findings for health professionals, policy makers and consumers and inform future clinical practice and research as the shift in genetic testing takes place.

Two previous reviews investigating studies into the process and content of genetic counselling concluded that genetic counselling is often provider-driven, educational and biomedical in content with little attention to psychosocial aspects [1, 2]. These two reviews addressed communication about all types of hereditary conditions, including women with and at risk of hereditary breast and ovarian cancer.

A review of the communication goals and needs of cancer patients identified unmet communication needs and concluded that communication outcomes are enhanced when health professionals attend to patients’ emotional needs [3]. Systematic reviews have highlighted the influence of health professionals’ personal characteristics on the effectiveness of communication with cancer patients [4, 5], the need for an individualised approach [6] and the importance of good communication skills [7].

No previous systematic or scoping reviews of communication about genetic testing or hereditary cancer management with breast and ovarian cancer patients were identified.

## Methods

### Scoping review methodology

This scoping review was informed by the methodology developed by key authors in the field [8–10]. The procedure was based on the most recent guidance for conducting scoping reviews [10, 11].

### Review question

The review question was driven by the population, concept and context of the review [11]: What is known about the communication that takes place about hereditary cancer between genetics or oncology health professionals and patients with breast or ovarian cancer?

### Search strategy

A search of MEDLINE, CINAHL, PsycINFO and EMBASE databases was undertaken for the following search terms adapted for each database: genetic counselling or genetic counseling or genetic testing and ovarian, breast or fallopian tube neoplasms or neoplastic syndromes, hereditary syndromes or hereditary breast and ovarian cancer syndrome or *BRCA1* or *BRCA2*. The databases were searched for studies published between 1994, when the *BRCA1* gene was identified, and October 2017. Forward and backwards citation searches were undertaken on the Web of Science and Scopus for included papers.

### Article selection

The inclusion and exclusion criteria used for screening the articles were based on the population, concept and context of the research question [10, 11] (Table 1). Titles and abstracts were screened by two independent researchers (CJ and CP) until a good level of agreement was reached (97%). Remaining articles were reviewed by CJ and validated by CP.

**Table 1:** Inclusion and exclusion criteria used for screening articles

	Included	Excluded
Population	• Women with a personal history of breast or ovarian cancer • Health professionals specialising in genetics or oncology, including genetic counsellors, geneticists, oncologists, surgeons and nurses	• Men with breast/prostate cancer • Cancers other than breast or ovarian cancer • Children • Individuals at risk
Concept	• Communication about genetic testing and/or hereditary cancer management • Process and/or content of verbal or written communication, the information needed, knowledge understood and recalled or the experience of this communication • Participants, contexts, interventions or strategies involving patients, health professionals, health conditions or communication methods	• Impact of genetic test results • Studies where findings for cancer patients were not presented separately from at-risk women • Family communication
Context	• Qualitative and quantitative original research articles of all designs published in English • Genetics or oncology setting • Communication after completing, prior to or during cancer treatment • Expected outcomes from literature about at-risk women: ○ Ratio of health professional–patient talk ○ Accuracy and extent of knowledge/recall ○ Met/unmet communication needs ○ Satisfaction ○ Distress/anxiety ○ Intention to have a procedure/investigation, for example, genetic testing/surgery ○ Experience/understanding ○ Communication methods	• Reviews, editorials, chapters and commentaries • Primary/palliative care setting

### Data extraction

Details of the method, participants, sample size and interventions and outcomes significant to the review question were documented onto a data extraction tool [11] by CJ. In accordance with scoping review methodology [8], quality assessment of selected studies was not undertaken.

### Data synthesis

The iterative process of data synthesis involved a preliminary synthesis of the study findings, exploration of relationships between the studies and summary of the synthesised findings in narrative form within the areas of study [12, 13]. To identify themes and develop a preliminary synthesis of the findings, the studies were organised according to the main area of study followed by identification of relationships between the studies.

## Results

### Overview of included studies

**Fig. 1:**
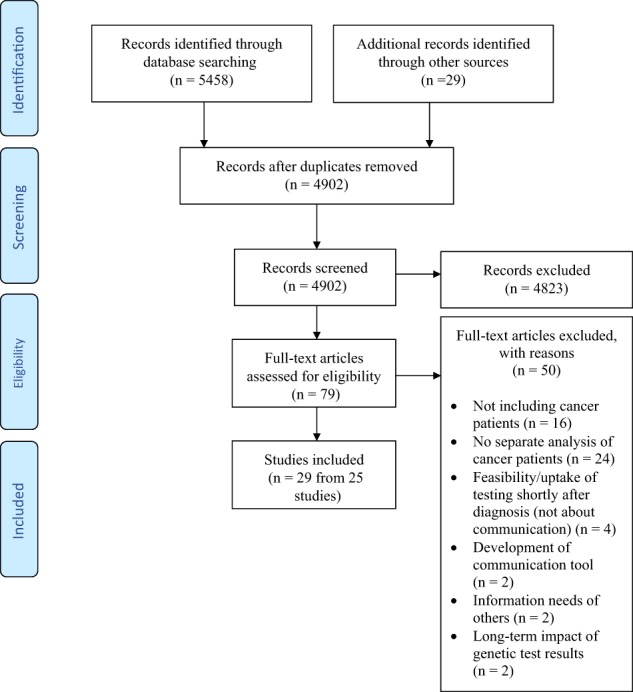
PRISMA flow diagram

The PRISMA flow diagram [14] shows the number of studies identified, included and excluded (Fig. 1). No studies of cancer patients were identified prior to 2000. Since 2008, more studies have focused on cancer patients only than on cancer patients and at-risk women combined. Health professionals were mainly genetic counsellors or clinical geneticists, although one study included the views of oncology health professionals. The included studies were published between 2000 and 2017 and were from the Australia, North America, Europe and Scandinavia. Most of the studies involved surveys. The types of participants, country of origin of the research and qualitative or quantitative research methods for the selected studies are shown in Table 2. Six areas of research were identified. Most studies investigated the cognitive and emotional impact of genetic counselling and testing. The research areas investigated by the selected studies are shown in Table 3. Details of the included studies are shown in Table 4.

**Table 2:** Types of participants, including patients and health professionals (HP), country/continent of research origin and qualitative/quantitative research methods in selected studies

References in ascending order according to year of publication—author [ref.]	Participants				Country/continent				Methods	
	Cancer patients and at-risk women	Cancer patients only	Genetics HPs	Oncology HPs	Europe/Scandinavia	UK	Australia	North America	Qual.	Quant.
Metcalfe et al. [16]	X							X		X
Randall et al. [23]		X					X			X
Hallowell et al. [35]		X				X			X	
Lobb et al. [21]^a^	X		X				X			X
Butow and Lobb [20]^a^	X		X				X			X
Lobb et al. [15]^a^	X		X				X			X
van Dijk et al. [36]	X				X					X
van Roosmalen et al. [39]	X				X					X
Mancini et al. [31]		X			X					X
Pieterse et al. [22]	X		X		X					X
Maheu and Thorne [34]		X						X	X	
Vadaparampil et al. [38]		X						X	X	
Vos et al. [37]		X			X				X	
Pieterse et al. [40]	X		X		X					X
Vadaparampil et al. [42]		X						X		X
Vos et al. [24]^b^		X			X					X
Christie et al. [27]		X						X		X
Meiser et al. [18]		X					X		X	
Vos et al. [25]^b^		X			X					X
Vos et al. [26]^b^		X			X					X
Gleeson et al. [17]		X					X		X	
Sie et al. [43]		X			X					X
Jacobs et al. [33]	X					X				X
Scherr et al. [29]		X						X		X
Quinn et al. [32]		X					X			X
Augestad et al. [41]		X			X				X	
Benusiglio et al. [30]		X			X					X
Bredart et al. [28]		X			X					X
Jacobs et al. [19]		X	X	X		X				X

^a^Papers from the same Australian study

^b^Papers from the same Netherlands study

**Table 3:** Areas of research

References in ascending order according to year of publication—author [ref.]	Information needs	Process and content	Cognitive and emotional impact			Experience	Timing of pre-test communication	Alternatives to face-to-face genetic counselling
			Recall and risk perception	Understand and interpret	Anxiety and distress			
Metcalfe et al. [16]	X							
Randall et al. [23]			X		X			
Hallowell et al. [35]				X		X		
Lobb et al. [21]^a^		X						
Butow and Lobb [20]^a^		X						
Lobb et al. [15]^a^	X	X						
van Dijk et al. [36]				X	X			
van Roosmalen et al. [39]					X			
Mancini et al. [31]			X		X			X
Pieterse et al. [22]		X						
Maheu and Thorne [34]				X		X		
Vadaparampil et al. [38]				X			X	
Vos et al. [37]				X		X		
Pieterse et al. [40]					X			
Vadaparampil et al. [42]							X	
Vos et al. [24]^b^			X					
Christie et al. [27]			X		X		X	
Meiser et al. [18]	X						X	
Vos et al. [25]^b^			X	X				
Vos et al. [26]^b^			X					
Gleeson et al. [17]	X						X	
Sie et al. [43]							X	X
Jacobs et al. [33]			X					
Scherr et al. [29]			X				X	
Quinn et al. [32]			X		X		X	X
Augestad et al. [41]						X	X	X
Benusiglio et al. [30]			X		X		X	X
Bredart et al. [28]			X		X			
Jacobs et al. [19]	X							

^a^Papers from the same Australian study

^b^Papers from the same Netherlands study

**Table 4:** Details of the included studies

References in ascending order according to year of publication—author [ref.]	Aims	Sample	Method	Findings relevant to cancer patients
Metcalfe et al. [16]	To identify the impact and information needs of women who undergo genetic counselling	79 participants—breast cancer patients (*n* = 46), at-risk women (*n* = 33)	Longitudinal survey	Cancer patients had unmet information needs about surgery, screening and chemoprevention
Randall et al. [23]	To investigate the psychological impact, including knowledge gained from genetic counselling and testing in women with breast cancer	64 breast cancer patients; genetic counselling group (*n* = 34), controls (*n* = 30)	Analysis of audiotaped genetic counselling consultations	There was no difference in psychological impact or knowledge gain between the groups
Hallowell et al. [35]	To investigate motivations for testing, information and support needs, and reactions to the test results	30 breast and ovarian cancer patients; carriers (*n* = 10) carriers, no pathogenic variant or VUS result (*n* = 12), awaiting results (*n* = 8)	Semi-structured qualitative interviews	The primary reason for testing was for family members. Waiting for results was not anxiety-provoking. Those who misinterpreted a result showing no pathogenic variant or VUS as good news were elated or relieved. Those who correctly interpreted the result as inconclusive felt disbelief, acceptance, disappointment, anger or frustration
Lobb et al. [21]^a^	To examine the influence of patients’ individual characteristics on health professionals’ behaviour during genetic counselling	158 participants—breast cancer patients (*n* = 69), at-risk women (*n* = 89); genetics health professionals (*n* = 7)	Analysis of audiotaped genetic counselling consultations	Genetics health professionals discussed more aspects of genetic testing, facilitated patient involvement and used more supportive and counselling behaviours with cancer patients than women at risk
Butow and Lobb [20]^a^	To describe the process and content of genetic counselling	As for Lobb et al. [21]	As for Lobb et al. [21]	Essential information was successfully communicated. Eliciting concerns and facilitating involvement was less successfully communicated. Risk information was infrequently communicated.
Lobb et al. [15]^a^	To investigate the effect of communication styles on patient outcomes	As for Lobb et al. [21]	As for Lobb et al. [21]	Cancer patients had unmet information needs about risk of contralateral breast cancer and risks for their relatives
van Dijk et al. [36]	To compare breast cancer risk and distress in women who receive different genetic test results	241 participants—breast cancer patients (*n* = 111), at-risk women (*n* = 130). VUS (*n* = 10), pathogenic variant (*n* = 34), negative predictive test (*n* = 37), no pathogenic variant or VUS (*n* = 160)	Longitudinal survey	There was no greater confusion or anxiety amongst the VUS group than the no pathogenic variant or VUS group. No differences were seen between the groups for understanding, perceived risk or distress after result
van Roosmalen et al. [39]	To evaluate the impact of *BRCA1/2* testing and disclosure of a positive test result on cancer patients and women at risk	89 participants - breast/ovarian cancer patients with a pathogenic variant (*n* = 23); at-risk women with a pathogenic variant (*n* = 66)	Randomised longitudinal survey	For both groups, anxiety-, depression- and cancer-related distress increased and general health decreased over time. Anxiety- and cancer-related distress was higher amongst cancer patients diagnosed ≤1 year than those tested ≥1 year. Intention to have risk-reducing surgery was higher among patients than at-risk women
Mancini et al. [31]	To assess the impact of a standardised information booklet on decision-making	560 breast cancer patients; Trial group (*n* = 297), controls (263)	Quasi-experimental trial, longitudinal survey	The booklet improved satisfaction, knowledge and decisional conflicts
Pieterse et al. [22]	To characterise breast cancer risk communication	51 participants—breast cancer patients (*n* = 34), at-risk women (*n* = 17): genetics health professionals (*n* = 10)	Longitudinal survey and video-recording of genetic counselling consultations	Most risks were communicated numerically or qualitatively and negatively. Patients’ preferred risk format and existing understanding was rarely sought. Cancer risks often were not communicated
Maheu and Thorne [34]	To explore the experience of women who receive genetic test result showing no pathogenic variant or VUS has been detected	21 breast and ovarian cancer patients	Semi-structured interviews	Results were shocking and difficult to interpret. Coping strategies included questioning the adequacy of testing, distrusting results and focusing on the similarities and differences with other families
Vadaparampil et al. [38]	To understand the experiences of breast cancer patients who have genetic counselling and testing prior to or after completing definitive cancer surgery.	9 breast cancer patients; tested prior to definitive treatment (*n* = 3), tested after definitive treatment (*n* = 6)	Semi-structured interviews	There were no differences in motivation for testing, influence of family on decision-making or expectations of testing between groups. Patients tested before surgery were unprepared for the implications of testing
Vos et al. [37]	To explain why cancer patients inaccurately perceive cancer risks when a VUS is detected	24 participants with a VUS—19 cancer patients - breast cancer (*n* = 17); ovarian cancer (*n* = 5); at-risk women (n = 5)	Semi-structured interviews and five-point Likert scales	Risk perception increased when a pathogenic variant was identified, VUS discussed pre-test, risk communicated in words, cancer perceived to be less severe and positive coping styles used
Pieterse et al. [40]	To evaluate outcomes of breast cancer genetic counselling in women with and without breast cancer	77 participants—breast cancer patients (*n* = 44), at-risk women (*n* = 33), genetics health professionals (*n* = 11)	Longitudinal survey and video-recording of genetic counselling consultations	Risk perception improved and anxiety was reduced in at-risk women. Risk perception unchanged and there was less reduction in anxiety for cancer patients
Vadaparampil et al. [42]	To evaluate satisfaction with the timing and strength of recommendations and information received prior to and during genetic counselling among breast cancer patients	51 breast cancer patients; genetic testing before definitive surgery (*n* = 25), genetic testing after definitive surgery (*n* = 26)	Survey	There was high satisfaction about the timing and strength of the recommendation for genetics referral. Patients had low levels of expectations pre-counselling and limited understanding of the process
Vos [24]^b^	To investigate accuracy of risk perception following genetic counselling	248 breast/ovarian cancer patients. Pathogenic variant (*n* = 30), VUS (*n* = 16), no pathogenic variant or VUS (*n* = 202)	Longitudinal survey	Medical decisions were influenced by perception of risk. The genetic test result and risk influenced outcomes
Christie et al. [27]	To investigate changes in cancer-related knowledge and emotional outcomes in women tested before and after definitive surgery	103 breast cancer patients counselled before (*n* = 16) and after (*n* = 87) definitive breast surgery	Longitudinal survey	Knowledge increased for both groups. Women tested before surgery showed deceased cancer -related stress and intrusive thoughts
Meiser et al. [18]	To identify information and communication preferences about genetic testing shortly after diagnosis for breast cancer	26 breast cancer patients diagnosed <50 years	Semi-structured qualitative interviews	There was a preference for personalised, brief, focused information. Specific information needs were highlighted
Vos et al. [25]^b^	To quantify the effect that perception has in genetic counselling for hereditary breast/ovarian cancer	As for Vos et al. [24]	As for Vos et al. [24]	Five years after genetic counselling recall of the result was accurate, but perception of the cancer risk and inheritance implications were inaccurate. A pathogenic variant and no pathogenic variant or VUS result were the only factors that predicted decisions about surgery and surveillance
Vos et al. [26]^b^	To investigate the short-term outcomes of communicating a genetic test result	As for Vos et al. [24]	As for Vos et al. [24]	Following genetic counselling risk management decisions were influenced by the perception of risk rather than by the communicated risks. The only counselling information that directly predicted counsellees’ perceptions and indirectly predicted outcomes were the DNA test result, the risk for the patient and the risk for her relatives
Gleeson et al. [17]	To identify information and communication preferences about genetic testing shortly after diagnosis for women with ovarian cancer	22 ovarian cancer patients	Semi-structured qualitative interviews	Patients expressed a preference for brief, positive, hope-giving information without statistics early in their diagnosis
Sie et al. [43]	To compare experiences of patients receiving pre-test information via written/digital formats with usual care	161 breast cancer patients; trial group (*n* = 95), usual care group (*n* = 66)	Survey	There were no differences in satisfaction, psychological distress, quality of life, breast cancer worry and risk perception for further cancer between groups
Jacobs et al. [33]	To compare the accuracy of information recall amongst patients and relatives following genetic counselling	32 participants - 10 breast and ovarian cancer patients and 22 of their at-risk relatives	Analysis of audiotaped genetic counselling consultations and post consultation interviews	71% of the information communicated during genetic counselling to cancer patients was about hereditary cancer management. Cancer patients accurately recalled 53% of the information
Scherr et al. [29]	To explore the impact of genetic counselling on breast cancer survivors’ knowledge about hereditary cancer over time	103 breast cancer patients; counselled before surgery (*n* = 16), counselled after surgery (*n* = 87)	Longitudinal survey	The knowledge gained following pre-test genetic counselling was not retained at 6 months
Quinn et al. [32]	To evaluate the efficacy of an educational pamphlet in preparing women for decision-making about genetic testing	136 breast cancer patients; Trial group (*n* = 66), controls (*n* = 70)	Randomised controlled non-inferiority trial, longitudinal survey	There were no differences between the groups for variance in knowledge and psychological outcomes
Augestad et al. [41]	To explore experiences of women with newly diagnosed cancer following genetic testing after written information only	17 breast and ovarian cancer patients	Semi-structured focus group interviews	The experience was shocking, distressing and overwhelming
Benusiglio et al. [30]	To evaluate group genetic counselling	210 breast and ovarian cancer patients	Longitudinal survey	Knowledge and satisfaction were increased over time
Bredart et al. [28]	To investigate the impact of genetic knowledge on feelings of personal control	243 breast cancer patients	Longitudinal survey	Breast cancer knowledge was not retained at post-test genetic counselling
Jacobs et al. [19]	To identify the key messages about *BRCA1/BRCA2* required by women with cancer	16 breast and ovarian cancer patients (service users) and 16 expert genetics and cancer health professionals	Delphi survey	Cancer patients agreed that 35 key messages should be communicated pre-test and post test. Of these, 30 key messages were agreed with health professionals. Disagreements were other cancers associated with *BRCA2*, diet and lifestyle and risks for non-carriers

^a^Papers from the same Australian study

^b^Papers from the same Netherlands study

### Areas of research

#### Patients’ information needs

Five articles were identified that addressed the information needs of cancer patients about hereditary cancer [15–19]. One study found that following pre-test genetic counselling, cancer patients (*n* = 69) reported unmet information needs about their risk of contralateral breast cancer and the cancer risks for their relatives [15]. Prior to counselling, 77% of the patients wanted information about their own risk and 98% wanted information about their relatives’ risk. This information was discussed in <45% of the consultations [15].

A survey of cancer patients (*n* = 46) and at-risk women (*n* = 33) found that cancer patients reported more unmet information needs about treatment options, including surgery, screening and chemoprevention [16]. Two qualitative studies, one with 26 breast cancer patients [18] and one with 22 ovarian cancer patients [17] investigated the actual and hypothetical information needs of those tested shortly after diagnosis. The studies identified a preference for brief, personalised, positive and straightforward information without statistics. Most patients considered it important to have information about the purpose of testing, the implications for treatment decisions, the time frame for results and the availability of predictive testing for relatives [17, 18]. A Delphi survey of 16 expert genetics and cancer health professionals and 16 service users with cancer and a *BRCA1/BRCA2* pathogenic variant agreed that information about inheritance, genetic testing, cancer risks and the management of hereditary cancer were key messages for cancer patients. The implications of genetic testing for treatment were not considered to be key messages [19].

#### Process and content of genetic counselling

Four articles from two studies addressed the process and content of genetic counselling communication [15, 20–22]. One of these studies [15, 20, 21] involved a content analysis of genetic counselling consultations with patients (*n* = 69) and at-risk women (*n* = 89) and seven genetics health professionals. Essential information about hereditary breast cancer was consistently communicated during pre-test counselling. Emotional concerns were not however always identified and genetics health professionals infrequently facilitated patients’ involvement in consultations [20]. The percentage of consultations in which specific cancer risks were discussed was similar for the cancer patients and the at-risk women [20]. Health professionals discussed significantly more aspects of genetic testing (*p* < .001), facilitated active patient involvement (*p* < .001) and used more supportive and counselling behaviours with cancer patients than with at-risk women (*p* = .02) [21]. Despite supportive counselling, 68% of the cancer patients studied did not feel reassured and 57% felt they could have been helped to cope better with their situation [15].

The same study also identified that the risk of a pathogenic variant was communicated in 13.3% of consultations with cancer patients undergoing diagnostic testing. With at-risk women, some of whom were undergoing predictive testing, the risk of a pathogenic variant was communicated in 71.8% of consultations [15]. With the cancer patients, the risk of further cancers in the presence of a pathogenic variant was discussed in 37.3% of consultations and the risk of further cancers in the *absence* of a pathogenic variant was discussed in 29.3% of consultations [20]. Information about their own and their relatives’ risks of cancer was communicated in <45% of consultations [15]. In a separate study involving a survey and analysis of transcripts of pre-test genetic counselling with 34 cancer patients and 17 at-risk women, the risk of contralateral or primary breast cancer was communicated to 38% of cancer patients and the risk of ovarian cancer to 50% [22].

The survey found that patients preferred risk to be personalised and presented in general terms [22]. However, risk was mostly communicated negatively in terms of harm, as a lifetime risk and numerically or qualitatively and health professionals rarely asked about preferred risk format or existing understanding of risk [22].

#### Cognitive and emotional impact of communication

##### Recall and risk perception

Eleven articles addressed recall and risk perception [23–33]. Of these, three articles were from the same study from the Netherlands [24–26]. Significant increase in knowledge was reported following pre-test communication by face-to-face genetic counselling [23, 27, 29], group education [28] and written communication [31, 32]. However, a significant reduction in hereditary cancer knowledge amongst breast cancer patients was identified between pre- and post-test genetic counselling [28, 29]. Low levels of accuracy of recall were observed amongst cancer patients in the weeks following genetic counselling [25, 33]. Similar findings were observed amongst breast cancer patients (*n* = 248) 5 years after genetic counselling when, although 75% of patients recalled their genetic test result, no more than 30% of patients accurately recalled the associated risks and likelihood of inheritance [25]. The same study found that mirroring risk reduced the accuracy of risk perception [24].

The only information provided by genetic counselling that predicted risk perception and risk management intentions concerned the genetic test result, the risk for the patient and the risk for relatives [26]. Cancer patients with a pathogenic variant who considered their cancer to be less severe and who used positive coping styles rather than avoidance strategies demonstrated more accurate risk perception than other patients [24].

##### Understanding and interpretation

Six studies addressed understanding and interpretation [25, 34–38]. Amongst patients found not to have a variant in a qualitative study, interpretation of the result varied, with some believing with certainty that they carried a pathogenic variant, some believing that they did not and some expressing uncertainty [34]. In a study of 248 patients receiving a pathogenic variant or no variant detected result directly predicted decisions about surgery or more frequent surveillance. All other decisions made as a result of the test were based on interpretation of risk rather than the actual communicated risk [25]. In a qualitative study, patients with a pathogenic variant were uncertain about which family members they should inform about their genetic risk [35].

A longitudinal survey found no difference in reported understanding of the result or perceived breast cancer risk between cancer patients who received a variant of uncertain significance (VUS) (*n* = 10) and those who did not have a variant (*n* = 37) [36]. In a further qualitative study of 17 patients with a VUS, 77% interpreted the result as pathogenic, despite factual recall of the result as not informative. Of these, 50% underwent risk-reducing surgery in the year following the result [37]. A qualitative study of 30 breast and ovarian cancer patients found that those who misinterpreted a VUS result as good news experienced elation or relief, whereas those who correctly understood the result as inconclusive experienced a range of emotions, including disbelief, acceptance, disappointment, anger or frustration [35]. Patients who adopted more positive coping styles had a better understanding of the risks associated with a VUS [37].

A qualitative study of breast cancer patients interviewed following pre-test genetic counselling prior to or after definitive surgery found a lack of understanding about what would be involved and misunderstanding about the inevitability of genetic testing. Patients were unaware even after genetic counselling of the utility of genetic testing and surprised that testing might result in further surgery and heightened emotions [38].

##### Anxiety and distress

Nine studies addressed anxiety and distress [23, 27, 28, 30–32, 36, 39, 40]. Pre-test communication did not have a negative impact on cancer-related distress, anxiety or depression, intrusive thoughts, decisional conflict, family involvement in decision-making or satisfaction amongst cancer patients in five of these studies [23, 27, 30–32]. However, three studies found that anxiety, depression and cancer-related distress were increased and general health decreased amongst cancer patients and at-risk women after disclosure of the genetic test result [28, 39, 40]. A longitudinal survey of 243 breast cancer patients found that amongst those who over-estimated their risk of a pathogenic variant at pre-test counselling, anxiety was raised at post-test counselling [28]. A randomised longitudinal survey found that cancer-related distress and anxiety was higher amongst patients diagnosed within a year of genetic testing than those tested over a year from diagnosis (*p* = .05) [39]. In a survey of breast cancer patients, no differences were found in anxiety or psychological distress between those who received a VUS and those who in whom no variant was detected [36].

### Patients’ experiences of communication

Four qualitative studies addressed patients’ experiences of communication [34, 35, 37, 41]. Three studies found that some patients tested after completing cancer treatment experienced confusion and shock upon receiving genetic test results [34, 35, 37]. A study of patients tested shortly after diagnosis without pre-test genetic counselling found that the emotional turmoil of the cancer diagnosis was heightened by the difficulty of receiving and comprehending the information [41]. Patients who were distressed by genetic test results that showed no variant was detected coped by questioning the adequacy of testing, distrusting the result and emphasising the difference between their family history and other higher risk families [34].

### Timing of pre-test communication

Ten studies addressed the timing of pre-test communication [17, 18, 27, 29, 30, 32, 38, 41–43]. Pre-test communication shortly after diagnosis was acceptable to most patients studied and did not cause distress [17, 18, 27, 29, 30, 32, 42, 43]. Some patients were unprepared for the implications of testing or did not understand the utility of the test [38, 42]. A qualitative study of 17 breast and ovarian cancer patients tested shortly after diagnosis without prior genetic counselling found that patients experienced shock, distress and confusion [41].

### Alternatives to face-to-face genetic counselling

Five studies addressed alternatives to face-to-face genetic counselling [30–32, 41, 43]. Knowledge and satisfaction were increased amongst patients who received pre-test communication via group education [30] or written information which aimed to supplement [30, 31] or replace genetic counselling [32]. Amongst 161 breast cancer patients who underwent genetic testing following written and digital communication without face-to-face genetic counselling, satisfaction with the amount and quality of pre-test information was high with most stating they would choose the same approach again and would recommend the approach to other patients [44]. No differences were found in psychological distress, quality of life or risk perception amongst patients who did and did not receive face-to-face pre-test genetic counselling [44]. A qualitative study with a small sample of these same patients however highlighted the need for support and counselling to increase understanding and empower decision-making [41].

## Discussion

Prior to 2008, most studies of communication about genetic testing for hereditary breast and ovarian cancer focused on at-risk women. Later studies of communication with cancer patients have mainly addressed genetic testing shortly after diagnosis. Although many studies have focused on the cognitive and emotional impact of genetic counselling, few have investigated patients’ information needs, the process and content of the communication or patients’ experiences of communication. Despite the increasing need to deliver genetic testing within mainstream oncology, all but one of the studies identified by this review involved genetics health professionals only and all addressed communication in the clinical genetics setting.

Although cancer patients expressed a need for simple, cancer-focused, personalised information, the information communicated about genetic testing is frequently difficult to understand [45] and does not always meet the needs of patients. For patients with cancer, information is often regarded as a method of reducing uncertainty and providing a sense of personal control as well as enabling informed decisions about treatment options [46, 47].

Genetic counselling of cancer patients focused on biomedical information-giving with less attention to psychological aspects. Studies of the process and content of genetic counselling with at-risk women have identified a similar focus [2, 45, 48–52]. In studies of communication by oncology health professionals with cancer patients, an individualised approach [6] and good communication skills [7] have been identified as requirements for effective communication.

Pre-test genetic counselling increased knowledge without raising anxiety or cancer-related distress. Similar findings have been observed following genetic counselling about hereditary breast and ovarian cancer with at-risk individuals [53, 54]. Amongst cancer patients, genetic counselling did not influence risk perception. In contrast, systematic reviews have concluded that genetic counselling improves risk perception in individuals at risk of breast cancer [2, 55] and other genetic conditions [56].

Genetic test results were misunderstood or misinterpreted, leading some patients to make inappropriate decisions about surgery and hindering family communication. Previous studies found that women with cancer understand their genetic risk in the context of their previous cancer experience [57] and identified lack of understanding of the risks and benefits of genetic information as a barrier to family communication [58, 59]. Amongst patients with a VUS, there were conflicting findings with some patients perceiving no difference in cancer risk and others perceiving the result to be a pathogenic variant. A lack of clear understanding about the meaning of a VUS has also been identified amongst breast cancer specialists [60].

There was an increase in anxiety, depression and cancer-related distress following results disclosure, especially amongst those who over-estimated their risk at pre-test genetic counselling and those tested without face-to-face genetic counselling. Increased cancer-related distress has also been observed in studies of the psychological impact of genetic testing amongst breast cancer patients following results disclosure [52, 57].

The few studies that investigated cancer patients’ experiences of communication found that for patients who were unprepared, unsupported or tested shortly after diagnosis without pre-test genetic counselling, the results disclosure was shocking, confusing and distressing. The impact of lack of support on experiences of genetic counselling has been highlighted for patients with and at risk of cancer [61, 62].

Communication about genetic testing shortly after diagnosis was acceptable for most patients. Early studies found high anxiety amongst women tested within 1 year of breast cancer diagnosis [63] and concern amongst health professionals and patients about the timing of testing [64]. However, studies investigating actual and hypothetical experiences of genetic testing shortly after diagnosis have found that, for the most part, patients and health professionals consider this type of genetic testing to be acceptable and desirable for decision-making if treatment options are improved as a result [65, 66].

For many cancer patients, pre-test communication without individual face-to-face genetic counselling may be acceptable, although this review identified that some patients need enhanced support and counselling. Other studies have found no differences in knowledge, distress or satisfaction amongst patients who have received genetics communication within the clinical genetics setting via group education [67] or video-conferencing [68].

This scoping review maps the current range of evidence specific to health professionals’ communication about hereditary cancer with cancer patients. This review should not be considered a definitive review of the literature in this field as, consistent with the methodology, no quality assessment was made. Due to the breadth of the studies reviewed, some areas of research only include a few studies. It is only possible to draw tentative conclusions about the clinical implications from these findings.

The findings suggest that greater attention may be needed to the psychological and supportive aspects of genetic counselling for cancer patients. If pre-test communication is increasingly to be provided via methods other than face-to-face genetic counselling, the post-test genetic counselling appointment may be the only opportunity for cancer patients to interact with a genetics health professional. Helping patients and families to adjust to a genetic diagnosis and facilitating dissemination of genetics information within families will become increasingly important aspects of the role of genetics health professionals. Patients with a pathogenic variant and those with a VUS may need additional help to understand the implications of genetic test results and support with decision-making about cancer management and cancer risk management. The limited studies of experiences of communication suggest that some patients may need enhanced counselling and support throughout the genetic testing process. As genetic testing becomes further integrated into mainstream oncology, it will be increasingly important to investigate communication about genetic testing by oncology health professionals and to develop and evaluate new models of communicating about genetic testing with cancer patients.

## Disclaimer

This article presents independent research funded by the NIHR. The views expressed are those of the authors and not necessarily those of the NHS, the NIHR or the Department of Health.
